# “Meaningful use” of electronic health records and its relevance to laboratories and pathologists

**DOI:** 10.4103/2153-3539.76733

**Published:** 2011-02-11

**Authors:** Walter H. Henricks

**Affiliations:** Center for Pathology Informatics, Pathology and Laboratory Medicine Institute, L21, Cleveland Clinic, 9500 Euclid Avenue, Cleveland, OH 44195, USA

**Keywords:** Electronic health records, federal regulations, laboratory information management, laboratory information systems, meaningful use

## Abstract

Electronic health records (EHRs) have emerged as a major topic in health care and are central to the federal government’s strategy for transforming healthcare delivery in the United States. Recent federal actions that aim to promote the use of EHRs promise to have significant implications for laboratories and for pathology practices. Under the HITECH (Health Information Technology Economic and Clinical Health) Act, an EHR incentive program has been established through which individual physicians and hospitals can qualify to receive incentive payments if they achieve “meaningful use” of “certified” EHR technology. The rule also establishes payment penalties in future years for eligible providers who have not met the requirements for meaningful use of EHRs. Meaningful use must be achieved using EHR technology that has been certified in accordance with functional and technical criteria that are set forth a regulation that parallels the meaningful use criteria in the incentive program. These actions and regulations are important to laboratories and pathologists for a number of reasons. Several of the criteria and requirements in the meaningful use rules and EHR certification criteria relate directly or indirectly to laboratory testing and laboratory information management, and future stage requirements are expected to impact the laboratory as well. Furthermore, as EHR uptake expands, there will be greater expectations for electronic interchange of laboratory information and laboratory information system (LIS)-EHR interfaces. Laboratories will need to be aware of the technical, operational, and business challenges that they may face as expectations for LIS-EHR increase. This paper reviews the important recent federal efforts aimed at accelerating EHR use, including the incentive program for EHR meaningful use, provider eligibility, and EHR certification criteria, from a perspective of their relevance for laboratories and pathology practices.

## INTRODUCTION

Electronic health record (EHR) systems are now a major topic in health care. Use of EHRs in physician practices and in healthcare organizations directly impacts the communication and management of laboratory information in patient care, particularly reporting of laboratory results and test order management. More pointedly, recent federal legislation and resultant regulations that aim to promote the use of EHRs promise to have substantial direct and indirect implications for laboratories and for pathology practice.

EHRs are central to the goals that the federal government has identified for improving healthcare:[1]

- Improve quality, safety, and efficiency of healthcare and reduce health disparities.
- Engage patients and families in their healthcare.
- Improve coordination of healthcare.
- Improve population and public health.
- Maintain privacy and security of health information.
- Reduce costs.

A minority of the physicians and healthcare organizations have fully implemented EHRs. Recent data from the CDC/National Center for Health Statistics indicate that 25% of the office-based physicians are using at least a “basic” EHR system, and only 10% are using a fully functional EHR.[2] In the most recent Healthcare Information Management Systems Society (HIMSS) Leadership Survey,[3] 22% of the healthcare organizations reported in 2010 that they had a fully operational electronic medical record across their entire organization (up from 17% in 2009), although only 5% reported that they had not yet begun to plan for electronic medical record implementation.

In recent months, the federal government has enacted regulations and programs in order to accelerate the implementation of EHRs by healthcare providers and healthcare organizations. This paper reviews the important recent federal efforts promoting EHR use, including regulations on EHR meaningful use and EHR certification criteria, and explores their relevance for laboratories and pathology practices.

## LEGISLATIVE AND REGULATORY BACKGROUND

The American Recovery and Reinvestment Act of 2009 (ARRA), enacted in February 2009, included several provisions that in aggregate comprise the Health Information Technology Economic and Clinical Health Act, or “HITECH Act.” The HITECH Act includes a number of provisions aimed at improving healthcare quality, safety, and efficiency through promotion of health information technology (HIT), notably EHRs, and through greater electronic exchange of health information. There are other aspects of the HITECH Act as well, including modifications to the HIPAA regulations; these other provisions are out of the scope of this paper, and the reader is referred elsewhere.[4]

**Figure 1:**
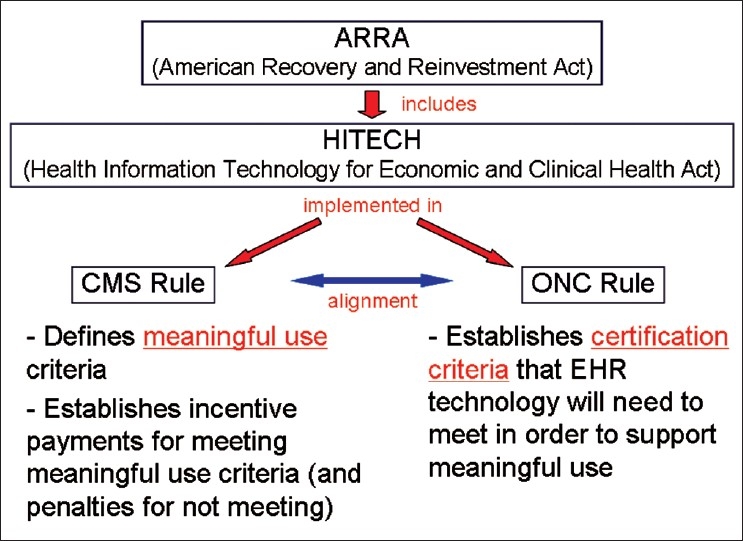
Relationships among ARRA, HITECH, and Final Rules from CMS and ONC. (CMS, Centers for Medicare and Medicaid Services; ONC, Office of the National Coordinator for Health Information Technology.)

A main goal of the HITECH Act is to foster meaningful use of certified EHR technology. Two recent, related major regulations have implemented the HITECH Act. Most significantly in this regard, the HITECH Act called for establishment of an incentive payment program for eligible professionals (e.g., physicians) and eligible hospitals that achieve “meaningful use” of qualified EHRs and interoperable HIT. To define and to implement this incentive program, in July 2010, Centers for Medicare and Medicaid Services (CMS) issued a Final Rule entitled Medicare and Medicaid Programs; Electronic Health Record Incentive Program (42 CFR Parts 412, 413, 422, *et al*).[5] The HITECH Act also required the Secretary of Health and Human Services (HHS) to adopt an initial set of standards, implementation specifications, and certification criteria for EHRs, along with establishing a certification program for EHRs. To meet these requirements, in July 2010, the Office of the National Coordinator for Health Information Technology (ONC) in the Department of Health and Human Services published a Final Rule entitled Health Information Technology: Initial Set of Standards, Implementation Specifications, and Certification Criteria for Electronic Health Record Technology (45 CFR Part 170).[6] The relationships among ARRA, HITECH, and these two regulations are depicted in Figure 1.

ONC is the primary agency in the federal government charged with developing and coordinating nationwide HIT policy and promoting the development of a nationwide health IT infrastructure for use and exchange of electronic health information. The ONC resides in the Department of Health and Human Services. ONC was created by Executive Order in 2004, and the HITECH Act made ONC permanent in law.

The two agencies in HHS, CMS and ONC, have worked together to coordinate the meaningful use criteria and the EHR certification criteria where appropriate. In short, EHR certification criteria (ONC-defined) specified “what” an EHR system must be able to do, while meaningful use criteria (CMS-defined) specified “how” a certified EHR system must be used by an eligible provider or within an eligible hospital environment to qualify for incentive payment and to avoid future penalties. Meeting criteria for meaningful use of EHR requires use of certified EHR technology.

## MEANINGFUL USE OF EHRs

### Overview

The EHR incentive program establishes the criteria, reporting requirements, incentive payments, and (future) penalties for eligible professionals and hospitals related to achieving the meaningful use of EHRs. Eligible professionals and eligible hospitals are those that participate in the Medicare or Medicaid programs. There are separate but related incentive programs for both Medicare and Medicaid-eligible providers and hospitals. Although there are some differences in some provisions of the administration of the Medicare and Medicaid programs, the meaningful use criteria and required quality measures are largely common to both. The Medicaid program will be voluntarily offered by individual states.

“Meaningful use” has no simple definition, and is ultimately defined by the specific requirements laid out in the Final Rule. The rule embodies and implements the statutory requirements of the HITECH Act that specified three requirements for meaningful use:[1]

- Use of certified EHR technology in a meaningful manner (e.g., e-prescribing).
- Use of certified EHR technology in a manner that provides for electronic exchange of health information to improve the quality of care.
- Use of certified EHR technology to submit clinical quality measures (CQM) and other measures determined by the HHS Secretary.

CMS intends to implement meaningful use requirements in three stages. The current rule describes Stage 1 requirements that are applicable to 2011 and 2012. CMS expects to update meaningful use criteria biannually, with Stage 2 criteria expected by the end of 2011 and Stage 3 criteria expected by the end of 2013. Stage 1 focuses essentially on capturing and sharing electronic health information at fundamental levels and establishing capabilities for data exchange and reporting data to various agencies. In Stage 2, CMS has indicated that it will build on the requirements of Stage 1 with more rigorous expectations for health information exchange and for additional EHR functionalities. In Stage 3, CMS expects to focus on promoting and making improvements that lead to improved health outcomes both at the individual and at the population levels, including greater use of decision support tools and patient access to self-management tools.

### Definitions, Eligibility, and Incentives/Penalties

The EHR incentive program Final Rule contains several intertwined definitions for different categories of EHR users. These definitions are fundamental to interpreting eligibility for incentives/penalties and the applicability of certain meaningful use requirements. The most relevant definitions of EHR users are:

Eligible Professional (EP) (sections 495.4, 495.100, 495.304):

For the Medicare EHR incentive program, EP generally includes the following types of professionals:

- Doctor of medicine or osteopathy.
- Doctor of dental surgery or medicine.
- Doctor of podiatric medicine.
- Doctor of optometry.
- Chiropractor.

For the Medicaid EHR incentive program, EP generally includes:

- Physician.
- Dentist.
- Nurse/mid-wife.
- Practitioner.
- Physician assistant in a federally qualified health center (QHC) or rural health center (RHC) that is so led by a physician assistant.

Hospital-based EP (495.4):

An EP (as defined under this section) who furnishes 90% or more of his or her covered professional services in a hospital setting in the year preceding the payment year.

Meaningful EHR user (495.4):

An EP (or) eligible hospital that, for an EHR reporting period for a payment year, demonstrates meaningful use of certified EHR technology.

Qualifying EP (Medicare-applicable) (495.100):

An EP who is a meaningful EHR user for the EHR reporting period for a payment year and who is not a hospital-based EP.

In the Medicare EHR incentive program, EPs can receive up to $44,000 in incentives over 5 years, although to get the maximum incentive payment the EPs must start participation by 2012. Note that only qualifying EPs will receive payments; in accordance with the definitions, qualifying EPs are those that me*et al*l the requirements for demonstrating meaningful use of certified EHR technology. Hospital-based EPs are not eligible to receive incentive payments. The stated rationale for excluding hospital-based EP from incentives is that paying incentives to hospital-based EPs and eligible hospitals would represent double payment, in that hospital-based EPs would be using the EHRs of the eligible hospitals. Under the Medicaid EHR incentive program, EPs can receive up to $63,750 over 6 years. Incentive payments for eligible hospitals are based on a number of factors, which include the number of acute care inpatient discharges and the number of inpatient bed-days. For both programs, eligible hospital incentive payments begin with a $2,000,000 base payment and may go up from there.

Beginning in 2015 and continuing in subsequent years, in the Medicare EHR Incentive Program, EPs who have not demonstrated meaningful use of certified EHR technology will receive reduced payments for professional services (495.102(d)). For 2015, the penalty will be a 1% reduction in the Medicare physician fee schedule amount for professional services, and this increases to 2% in 2016 and to 3% for 2017 and each subsequent year. Hospital-based EPs are not subject to the fee schedule reduction penalties that start in 2015. No payment reductions are included in the Medicaid EHR incentive program.

As described above, hospital-based EPs are not eligible for EHR meaningful use incentive payments and are not subject to downward payment adjustment penalties for not being meaningful users of EHRs. The definition and means of determining a “Hospital-based EP” are clarified in the Provisions of the Proposed Rule and Analysis of and Responses to Public Comment in pages 44439–44442 of the CMS Final Rule. CMS defines a hospital-based EP as an EP who furnishes 90% or more of his/her covered professional services in a hospital setting in the year preceding the payment year. A setting is considered a hospital setting if it is a site of service that would be identified by the codes used in the HIPAA Standard Transaction as a hospital inpatient or emergency room setting. Specifically in the Final Rule, CMS indicates that it will use only two place of service (POS) codes used on physician claims to determine whether an EP is a hospital-based EP: POS 21 (Inpatient Hospital) or POS 23 (Emergency Room, Hospital). From the rule:

“An EP will be defined as being hospital-based and therefore ineligible to receive an EHR incentive payment under either Medicare or Medicaid, regardless of the type of service provided, if more than 90 percent of their services are identified as being provided in places of service classified under two place of service codes 21 (Inpatient Hospital) or 23 Emergency Room, Hospital.”

### Are Pathologists Subject to Future Meaningful Use Penalties?

The definition of hospital-based EP in the Final Rule raises questions and concerns as to whether pathologists are eligible for incentives and, more importantly, subject to penalties for not being meaningful users of EHR. The Core Measures and CQM that are required to become a Meaningful User of Certified EHR technology (see later section) are largely either not applicable to or out of the scope of the practice of pathology. Furthermore, pathologists (generally) do not see and treat patients in an office setting and, therefore, the use of certified EHR technology in the comprehensive manner prescribed by CMS is not relevant to the pathologists.

Any eligible provider who is not either a qualifying EP (i.e., meaningful user of an EHR) or a hospital-based EP will be subject to Medicare payment reductions starting in 2015. Part of the issue stems from the fact that the definition of “hospital-based” is rooted in the law and a subsequent amendment of the HITECH Act. The CMS Final Rule explains (italics added):

“Sections 4101(a) and 4201(a) of the HITECH Act originally defined the term ‘hospital-based eligible professional’ to mean an EP, such as a pathologist, anesthesiologist, or emergency physician, who furnishes substantially all of his or her Medicare-covered professional services during the relevant EHR reporting period in a hospital setting (*whether inpatient or outpatient*) through the use of the facilities and equipment of the hospital, including the hospital’s qualified EHRs.”

In April 2010, however, after the publication of the incentive program Interim Final Rule that was available for public comment, an amendment to the HITECH Act was signed into law that changed the statutory definition of a hospital-based EP. This amendment changed the key wording in the definition from “…in a hospital setting (*whether inpatient or outpatient*)…” to “*in a hospital inpatient or emergency room setting*”.

The removal of “outpatient” is crucial to the relevance of the new definition of hospital-based EP to pathologists because, typically, greater than 10% of the services (physician claims) that pathologists provide, even pathologists in hospital settings, are for outpatients. Based on the new definition of hospital-based EP, the method for determining whether an EP is hospital-based is based “solely” on the POS codes, on physician claims being POS 21 (Inpatient Hospital) or POS 23 (Emergency Room, Hospital).

Pathologists’ concerns about being subject to payment penalties because the requirements for Core Measures and CQM are outside the scope of pathology practice were addressed directly in the Final Rule. In brief, CMS indicated that the definition of hospital-based EP is based in law and that the Secretary (of HHS) has no discretion to exempt pathologists from the definition in the law (p.44443):

“An organization representing pathologists expressed concern that the Medicare EP definition, as currently drafted would subject certain pathologists to payment incentive penalties for not being meaningful EHR users if the pathologists performed less than 90 percent of their professional services in any inpatient or outpatient setting in the prior year. All EPs have to report on all Core Measures and a subset of clinical measures that pathologists could not meet in their day-to-day practice given the nature of pathology’s scope of practice. Accordingly, this organization recommended that CMS ensure that pathologists who are currently defined as Medicare EPs be considered as ‘non-qualifying’ EPs, that are exempt from future meaningful user penalties.

*Response* (from CMS; italics added): While we appreciate the comments that we received on the Medicare EP definition, *we are unable to expand or alter this statutory definition* or consolidate it with the Medicaid program EP definition as suggested by the commenters. Under the EHR incentive payment program, *the law provided a separate Medicare EP definition rather than giving the Secretary authority or discretion to determine who is a Medicare EP* or, who is an EP for both the Medicare and Medicaid programs.”

The legal definition of hospital-based EP and the manner in which it will be determined appear to indicate that pathologists for whom less than 90% of professional services fall under POS codes for hospital inpatient (POS 21) or emergency room (POS 23) will not meet the definition of hospital-based EP that would exempt them from future meaningful use penalties. While further clarification would be welcome, pathology practices should be aware of these issues and should assess their situations in light of the combination of the eligibility considerations and definitions described above and the lack of applicability of EHR meaningful use requirements to general pathology practice.

## MEANINGFUL USE REQUIREMENTS

The CMS Final Rule on the EHR incentive program lays out the requirements for eligible professionals and eligible hospitals to meet the definition of meaningful use of certified EHR technology. The requirements are a combination of required core objectives, objectives selected from a menu set, and reporting of CQMs in a manner specified by the HHS Secretary.

Eligible professionals must meet:

- 15 core objectives.
- Five objectives out of a menu set of 10.
- Reporting requirements for six CQMs.
- Three core or alternate core CQMs and three of 38 from an additional set of CQMs.

Hospitals must meet:

- 14 core objectives.
- Five objectives out of a menu set of 10.
- Reporting requirements for 15 CQMs.

The core objectives for eligible providers are listed in Table 1 and for eligible hospitals in Table 2. The menu set objectives for eligible providers are listed in Table 3 and for eligible hospitals in Table 4. Many of the Stage 1 objectives will have compliance assessed by obtaining a certain percentage measure. For instance, to meet certain objectives, 80% of patients must have records in certified EHR technology. Other objectives are related to the presence or absence of certain functions such as implementation of at least one clinical decision support rule.

**Table 1:** Core objectives for meaningful use of EHRs: Eligible professionals

Computerized provider order entry (CPOE) E-prescribing (eRx) Report ambulatory Clinical Quality Measures to CMS/States Implement one clinical decision support rule Provide patients with an electronic copy of their health information, upon request** Provide clinical summaries for patients for each office visit Drug–drug and drug–allergy interaction checks Record demographics Maintain an up to date problem list of current and active diagnoses Maintain active medication list Maintain active medication allergy list Record and chart changes in vital signs Record smoking status for patients 13 years or older Capability to exchange key clinical information among providers of care and patient-authorized entities electronically** Protect electronic health information

** Objectives that specifically mention laboratory results or diagnostic test results; EHRs - Electronic health records. (Source: Medicare and Medicaid EHR Incentive Program Meaningful Use Stage 1 Requirements Overview at www.cms.gov/EHRIncentivePrograms/Downloads/MU_Stage1_ReqOverview.pdf; reference[1])

**Table 2:** Core objectives for meaningful use of EHRs: Hospitals

Computerized provider order entry (CPOE) Drug–drug and drug–allergy interaction checks Record demographics Implement one clinical decision support rule Maintain up-to-date problem list of current and active diagnoses Maintain active medication list Maintain active medication allergy list Record and chart changes in vital signs Record smoking status for patients 13 years or older Report hospital Clinical Quality Measures to CMS or States Provide patients with an electronic copy of their health information, upon request** Provide patients with an electronic copy of their discharge instructions at time of discharge, upon request Capability to exchange key clinical information among providers of care and patient-authorized entities electronically** Protect electronic health information

* Objectives that specifically mention laboratory results or diagnostic test results; EHRs - Electronic health records. (Source: Medicare and Medicaid EHR Incentive Program Meaningful Use Stage 1 Requirements Overview at www.cms.gov/EHRIncentivePrograms/Downloads/MU_Stage1_ReqOverview.pdf; reference[1])

**Table 3:** Menu objectives for meaningful use of EHRs: Eligible professionals***

Drug-formulary checks Incorporate clinical lab test results as structured data** Generate lists of patients by specific conditions Send reminders to patients per patient preference for preventive/follow-up care Provide patients with timely electronic access to their health information** Use certified EHR technology to identify patient-specific education resources and provide to patient, if appropriate Medication reconciliation Summary of care record for each transition of care/referrals Capability to submit electronic data to immunization registries/systems* Capability to provide electronic syndromic surveillance data to public health agencies*

* At least one public health objective must be selected);

** Objectives that specifically mention laboratory results or diagnostic test results; EHRs - Electronic health records. (Source: Medicare and Medicaid EHR Incentive Program Meaningful Use Stage 1 Requirements Overview at www.cms.gov/EHRIncentivePrograms/Downloads/MU_Stage1_ReqOverview.pdf; reference[1])

*** Eligible professionals must choose five menu objectives; may defer five of 10;

**Table 4:** Menu objectives for meaningful use of EHRs: Hospitals***

Drug-formulary checks Record advanced directives for patients 65 years or older Incorporate clinical lab test results as structured data** Generate lists of patients by specific conditions Use certified EHR technology to identify patient-specific education resources and provide to patient, if appropriate Medication reconciliation Summary of care record for each transition of care/ referrals Capability to submit electronic data to immunization registries/systems* Capability to provide electronic submission of reportable lab results to public health agencies* Capability to provide electronic syndromic surveillance data to public health agencies*

* At least one public health objective must be selected;

** Objectives that specifically mention laboratory results or diagnostic test results; EHRs - Electronic health records. (Source: Medicare and Medicaid EHR Incentive Program Meaningful Use Stage 1 Requirements Overview at www.cms.gov/EHRIncentivePrograms/Downloads/MU_Stage1_ReqOverview.pdf; reference[1])

*** Hospitals must choose five menu objectives; may defer five of 10;

CQMs are assessments and measures of healthcare quality that have been developed and endorsed by CMS in collaboration with other healthcare quality agencies such as the National Quality Forum (NQF), the Agency for Healthcare Research and Quality (AHRQ), and others. With respect to reporting CQMs to CMS (or to the State in the case of the Medicaid program), the Final Rule indicates that CQMs are to be reported in the manner specified by CMS. CMS specifies the CQMs for eligible professionals in Table 6 of its final rule, and specifies core and alternate core measures in Table 7 of its final rule. CQMs for hospitals are listed in Table 10 of the final rule. The CQMs are summarized here in Tables 5 and 6. Eligible professionals must report on a total of 6 CQM, of which three must be from a defined core set of three CQMs or from a set of up to three alternate core CQMs if one or more core set CQM(s) is not applicable to a given eligible professional’s practice setting. In addition, eligible providers must report on three CQMs out of a menu of 38 options [Table 5]. Overall, eligible professionals must report on a total of six CQMs – three core or alternate core measures and three additional measures. Hospitals must report on 15 quality measures [Table 6].

**Table 5:** Core Set CQMs (must complete three core or alternate core)

Hypertension: blood pressure measurement Preventive care and screening measure pair: (a) tobacco use assessment, (b) tobacco cessation intervention Adult weight screening and follow-up
Alternate core set CQMs
Weight assessment and counseling for children and adolescents Preventive care and screening: Influenza immunization for patients 50 years old or older Childhood immunization status
Additional set CQM (must complete three of 38)
Diabetes: hemoglobin Alc poor control* Diabetes: low-density lipoprotein (LDL) management and control* Diabetes: blood pressure management Heart failure (HF): angiotensin-converting enzyme (ACE) inhibitor or angiotensin receptor blocker (ARB) therapy for left ventricular systolic dysfunction (LVSD) Coronary artery disease (CAD): beta-blocker therapy for CAD patients with prior myocardial infarction (MI) Pneumonia vaccination status for older adults Breast cancer screening Colorectal cancer screening* Coronary artery disease (CAD): oral antiplatelet therapy prescribed for patients with CAD Heart failure (HF): beta-blocker therapy for left ventricular systolic dysfunction (LVSD) Anti-depressant medication management: (a) effective acute phase treatment, (b) effective continuation phase treatment Primary open angle glaucoma (POAG): optic nerve evaluation Diabetic retinopathy: documentation of presence or absence of macular edema and level of severity of retinopathy Diabetic retinopathy: communication with the physician managing ongoing diabetes care Asthma pharmacologic therapy Asthma assessment Appropriate testing for children with pharyngitis* Oncology breast cancer: hormonal therapy for Stage IC–IIIC estrogen receptor/progesterone receptor (ER/PR) positive breast cancer* Oncology colon cancer: chemotherapy for Stage III colon cancer patients Prostate cancer: avoidance of overuse of bone scan for staging low-risk prostate cancer patients Smoking and tobacco use cessation, medical assistance: (a) advising smokers and tobacco users to quit, (b) discussing smoking and tobacco use cessation medications, (c) discussing smoking and tobacco use cessation strategies Diabetes: eye exam Diabetes: urine screening* Diabetes: foot exam Coronary artery disease (CAD): drug therapy for lowering LDL-cholesterol Heart failure (HF):Warfarin therapy patients with atrial fibrillation Ischemic vascular disease (IVD): blood pressure management Ischemic vascular disease (IVD): use of aspirin or another antithrombotic Initiation and engagement of alcohol and other drug dependence treatment: (a) initiation, (b) engagement Prenatal care: screening for human immunodeficiency virus (HIV)* Prenatal care: anti-D immune globulin* Controlling high-blood pressure Cervical cancer screening* Chlamydia screening for women* Use of appropriate medications for asthma Low-back pain: use of imaging studies Ischemic vascular disease (IVD): complete lipid panel and LDL control* Diabetes: hemoglobin Alc control (<8.0%)*

* CQMs that include or depend on laboratory testing; CQMs - Clinical quality measures; EHRs - electronic health records. (Source: Medicare and Medicaid EHR Incentive Program Meaningful Use Stage 1 Requirements Overview at www.cms.gov/EHRIncentivePrograms/Downloads/MU_Stage1_ReqOverview.pdf; reference[1])

**Table 6:** CQMs for meaningful use of EHRs: Hospitals (must complete all 15)

Emergency department throughput – admitted patients median time from ED arrival to ED departure for admitted patients Emergency department throughput – admitted patients –admission decision time to ED departure time for admitted patients Ischemic stroke – discharge on antithrombotics Ischemic stroke – anticoagulation for A-fib/flutter Ischemic stroke – thrombolytic therapy for patients arriving within 2 h of symptom onset Ischemic or hemorrhagic stroke – antithrombotic therapy by day 2 Ischemic stroke – discharge on statins* Ischemic or hemorrhagic stroke – stroke education Ischemic or hemorrhagic stroke – rehabilitation assessment VTE prophylaxis within 24 h of arrival Intensive care unit VTE prophylaxis Anticoagulation overlap therapy* Platelet monitoring on unfractionated heparin* VTE discharge instructions Incidence of potentially preventable VTE

* CQMs that include or depend on laboratory testing; CQMs - Clinical quality measures; EHRs - electronic health records. (Source: Medicare and Medicaid EHR Incentive Program Meaningful Use Stage 1 Requirements Overview at www.cms.gov/EHRIncentivePrograms/Downloads/MU_Stage1_ReqOverview.pdf; reference[1])

**Table 7:** EHR certification criteria that mention laboratory results as part of the functional requirement (42 CFR 170.302, 304, 306)

Incorporate laboratory test results (general criterion) Generate patient lists (laboratory results as criterion) (general criterion) Computerized provider order entry (CPOE) (ambulatory, inpatient) Patient reminders (ambulatory) Clinical decision support – implement rules (ambulatory, inpatient) Electronic copy of health information* (ambulatory, inpatient) Timely access (for patients) (ambulatory) Clinical summaries* (ambulatory) Exchange clinical information and patient summary record* (ambulatory, inpatient)

* Criteria specifically referencing LOINC as a requirement for representing laboratory results; EHR - electronic health record

CMS finalized as reporting requirements only those CQMs for which there are available electronic specifications (as of the date of the Final Rule). CMS indicates in the Final Rule that additional CQM will be included in proposed Stage 2 meaningful use requirements.

### Meaningful Use Requirements Most Applicable to Laboratories

The meaningful use requirement most directly relevant to laboratories is one from the menu set objectives:

“More than 40% of all clinical lab tests results ordered by the EP or by an authorized provider of the eligible hospital…whose results are either in a positive/negative or numerical format are incorporated in certified EHR technology as structured data.”

Regarding the definition of “structured” data in this context, the Final Rule states that (p.44346):

“Structured data is not fully dependent on an established standard…Structured data within certified EHRs technology merely requires the system to be able to identify the data as providing specific information. This is commonly accomplished by creating fixed fields within a record on file but not solely accomplished in this manner.”

While CMS highly encourages electronic data exchange of laboratory results, the measure does not include a specific requirement for transmission or electronic receipt of lab results (although such a requirement is expected in future stages). Meeting the above requirement, however, in most settings will be realistically possible only with an electronic interface between the laboratory information system (LIS) and the EHR (rather than through manual entry).

Some of the core and menu set meaningful use objectives include laboratory test results as part of the required data elements. The meaningful use core objectives and menu set objectives that specifically mention laboratory test results or diagnostic test results in the rule are denoted in Tables 1–4. In addition, several of the ONC EHR certification criteria (see below) specify functional requirements that include handling of laboratory and/or diagnostic test results in EHRs. Some of these certification requirements that involve laboratory results underpin meaningful use objectives in the CMS Final Rule that may not mention the laboratory results specifically. Examples include EHR capabilities to implement decision support rules based on laboratory results and to provide patients with online access to clinical information that includes laboratory results.

Twelve of the CQMs in the Stage 1 meaningful use requirements for eligible professionals include measures that include or depend upon laboratory testing. For example, the CQM entitled “Diabetes: Hemoglobin A1c Poor Control” requires reporting of the percentage of patients between 18 and 75 years old with diabetes (type 1 or 2) who had hemoglobin A1c greater than 9.0%. Three of the reportable CQMs required of hospitals include or depend upon laboratory testing. The CQMs that involve laboratory testing are denoted in Tables 5 and 6.

Computerized Physician Order Entry (CPOE) for laboratory test orders was a requirement that was initially included in the CMS-proposed Interim Final Rule; however, the CMS chose to remove the CPOE requirements for laboratory test requirements for Stage 1 in the Final Rule. It is made clear in the Final Rule, however, that CMS expects to include CPOE requirements for laboratory tests in Stage 2. In addition, a CPOE requirement for laboratory test orders are specifically included in the current ONC EHR certification criteria.

## ONC CERTIFICATION CRITERIA FOR EHRs

In July 2010, the Department of Health and Human Services and the Office of the National Coordinator for Health Information Technology (ONC) published the Final Rule: Health Information Technology: Initial Set of Standards, Implementation Specifications, and Certification Criteria for Electronic Health Record Technology (45 CFR Part 170).[6] This rule identifies the functional and technical capabilities that the EHR technology and systems must possess and demonstrate in order to ensure that uses can use such technology to achieve Stage 1 meaningful use criteria:

“…certification criteria establish the required capabilities and specify the related standards and implementation specifications that serve as an electronic health record (EHR) technology will need to include to, at a minimum, support the achievement of meaningful use Stage 1 by eligible professionals, eligible hospitals, and/or critical access hospitals…under the Medicare and Medicaid EHRs Incentive Programs.”

The Final Rule sets forth the following definitions of certified EHRs technology (170.102):

“*Certified EHR Technology* means: (1) A Complete EHR that meets the requirements included in the definition of a Qualified EHR and has been tested and certified in accordance with the certification program established by the National Coordinator as having met all applicable certification criteria adopted by the Secretary; or

(2) A combination of EHR Modules in which each constituent EHR Module of the combination has been tested and certified in accordance with the certification program established by the National Coordinator as having met all applicable certification criteria adopted by the Secretary, and the resultant combination also meets the requirements included in the definition of a Qualified EHR.

*Complete EHR* means EHR technology that has been developed to meet, at a minimum, all applicable certification criteria adopted by the Secretary.”

Elsewhere in the Final Rule, ONC defined an EHR Module and added clarification as follows:

“…‘any service, component, or combination thereof that can meet the requirements of at least one certification criterion adopted by the Secretary.’ Consequently, EHR Modules, by definition, must provide a capability that can be tested and certified in accordance with at least one certification criterion adopted by the Secretary.”

and,

“An EHR Module could provide a single capability required by one certification criterion or it could provide all capabilities but one, required by the certification criteria for a Complete EHR.”

The ONC rule includes certification criteria that are applicable generally to EHRs and criteria that are applicable more specifically to EHRs designed for ambulatory and for inpatient settings. While the ONC certification criteria align with and complement the meaningful use requirement in the CMS EHR incentive programs, the distinction between the CMS and ONC rules is important to understanding and interpreting the regulations. The ONC rule describes the capabilities that certified EHR technology must be able to demonstrate to support the use of the EHR in a manner that meets the meaningful use objectives of the CMS rule. Certification criteria also require that EHR technology can generate reports for each meaningful use objective measure that is percentage based (including numerator, denominator, and percentage). The specified capabilities include compliance with data standards in certain circumstances or for particular functions. The ONC rule makes a point that the rule is not intended to specify when or how persons or organizations using EHR technology must implement particular capabilities in their environments. Rather, the “how” of using EHRs is the purview of current and future meaningful use requirements:

“…we anticipate that future meaningful use objectives and measures will specify, as necessary and appropriate, the conditions which certain health care providers will need to use adopted standards and implementations specifications.”

### Certification Criteria and Standards Most Relevant to Laboratories

Several of the certification requirements for EHRs specifically mention laboratory and/or diagnostic test results, and some of these align with meaningful use objectives that may not overtly specify laboratory results. EHR certification criteria that specifically mention laboratory results as part of the EHR functional requirements are listed in Table 7.

The certification criterion that most directly relates to laboratory testing is a requirement for EHR technology in general in 170.302:

(h) *Incorporate laboratory test results—(1) Receive results.*

Electronically receive clinical laboratory test results in a structured format and display such results in a human readable format.

(2) *Display test report information.*

Electronically display all the information for a test report specified at 42 CFR 493.1291(c)(1) through (7).

(3)* Incorporate results.*

Electronically attribute, associate, or link a laboratory test result to a laboratory order or patient record.

This certification criterion only states that EHRs must be able to receive laboratory results in a structured format. The rule does not impose any further or specific requirements for what constitutes a “structured” format in this context, stating:

“…we do not believe that it is within the scope of this rule to dictate the standard by which laboratories transmit test results.”

The ONC certification requirement correlates with the CMS meaningful use requirement, which states basically that 40% of clinical lab test results whose results are either in a positive/negative or numerical format are incorporated into EHR technology as structured data. As described earlier, the description of structured data in the CMS rule on meaningful use states that “structured data within certified EHR technology merely requires the system to be able to identify the data as providing specific information.”

The only requirement relating to the content and manner for laboratory results display in the certification criterion above is that EHRs display elements that are specified in the CLIA rule (42 CFR 493.1291 (c)(1) through (7)), which states:

(c) The test report must indicate the following:

1. For positive patient identification, either the patient’s name and identification number or a unique patient identifier and identification number.
2. The name and address of the laboratory location where the test was performed.
3. The test report date.
4. The test performed.
5. Specimen source, when appropriate.
6. The test result and, if applicable, the units of measurement or interpretation, or both.
7. Any information regarding the condition and disposition of specimens that do not meet the laboratory’s criteria for acceptability.

No other requirements pertaining to how laboratory result data are displayed in EHRs are included in the certification criteria.

Another EHR certification criterion that relates specifically to laboratory testing includes the capability for CPOE for laboratory orders, both in ambulatory and in inpatient EHRs. The difference between this requirement and the CPOE requirement in the meaningful use objectives may be easily confused. To clarify, this requirement for CPOE for laboratory orders is a capability that an EHR must possess to become certified. This is in distinction to the meaningful use criteria, in which the use of CPOE for laboratory orders is not required for Stage 1. This certification criterion paves the way for the expected requirements of CPOE for laboratory orders in Stage 2 of meaningful use.

### Data Standards for Electronic Health Information, Including LOINC

In addition to the certification requirements for EHRs technology to support meaningful use in Stage 1, the Final Rule sets forth the HIT standards that have been deemed to have been adopted by the Secretary. Standards designated in the rule include:

- Content exchange standards, including HL7 version 2.5.1 and/or version 2.3.1 for certain public health data reporting requirements (170.205).
- Vocabulary standards for representing electronic health information, including ICD-9-CM, SNOMED CT, and Logical Observation Identifiers Names and Codes (LOINC) (170.207).
- Standards for protecting the exchange of electronic health information, including encryption standards (170.210).

The deemed standards are in turn referenced as part of specific certification requirements and implementation specifications. For instance, ambulatory EHR systems must enable a user to create an electronic copy of a patient’s clinical information that includes a problem list (170.304(f)) that uses either vocabulary standard ICD-9-CM or SNOMED CT. Another example is the requirement for inpatient EHR systems to submit reportable lab results to public health agencies (170.306(g)), which requires the use of HL7 v2.5.1 for that particular criterion.

LOINC is specified in the ONC rule as a vocabulary standard for representing laboratory test results; however, it is important to realize that the requirement in the regulation regarding LOINC at this time is that certified EHR technology must be able to re-use a LOINC when it has been received from the laboratory and such code is accessible in the EHR. Specifically, the rule states that the HHS Secretary adopts LOINC as a standard for laboratory test results (only) “when such codes were received within an electronic transaction from a laboratory.” In other words, when received from a laboratory as LOINC codes, the EHRs must be able to use those LOINC codes for other certification criteria in which use of laboratory data is required, such as electronic copies of health information for patients (170.304(f)), clinical summaries (170.304(h)), and others. The EHR certification criteria that specifically reference LOINC as a requirement are noted as part of Table 7. It is worth reiterating here that neither the current ONC certification criteria and implementation specifications nor the Stage 1 CMS meaningful use requirements require laboratories to transmit results using LOINC codes.

### ONC Temporary Certification Program for EHR Technology

Elsewhere in the ONC’s Final Rule (45 CFR Part 170 subpart D), in June 2010, the ONC established a Temporary Certification Program for EHR Technology. This program authorizes ONC-Authorized Testing and Certification Bodies (ONC-ATCBs) to test EHR technology and to certify that EHR systems meet the standards, implementation specifications, and certification criteria as specified in the Final Rule. The rule also describes how organizations can become ONC-ATCBs. The temporary certification program will be replaced eventually by a permanent certification program. As of this writing, three organizations have qualified as ONC-ACTBs:

- Certification Commission for Health Information Technology (CCHIT).
- Drummond Group Inc. (DGI).
- InfoGard Laboratories Inc.

An up to date list of ONC-ATCBs may be found on the ONC web site at http://healthIT.hhs.gov/ATCBs. ONC maintains an up to date Certified HIT Product List (CHPL) at http://onc-chpl.force.com/ehrcert. As of this writing, the CHPL web site lists 115 systems from 79 vendors that have been certified by ONC-ATCBs under the Temporary Certification Program.

### Other Related ONC Programs

There are two other ONC-sponsored programs (from HITECH) of which pathologists and laboratories should be aware, which have been created to foster EHRs adoption: Regional Extension Centers (RECs) and Health Information Exchanges (HIEs).

The HITECH Act allocated $677 million to the establishment of Health Information Technology Regional Extension Centers (RECs) that will offer health care providers with technical assistance, guidance, and information on best practices to support and accelerate health care providers efforts to become meaningful users of EHRs.[7] RECs cover all geographic regions of the United States. As of this writing, 62 RECs for practitioners have had funding announced and an additional 46 RECs have been funding to help critical access and rural hospitals adopt certified EHR technology. The RECs will focus mostly on clinicians providing primary care services, with an emphasis on individual and small group practices (fewer than 10 providers). The RECs aim to provide assistance in EHRs product selection and implementation as well as guidance on improving clinical administrative workloads to use EHRs most effectively, and meeting legal, regulatory, and other requirements. The relevance of RECs to laboratories is that understanding RECs efforts in their area may provide an opportunity for laboratories to work with the RECs, physician offices, and EHR vendors to improve success in implementing laboratory interfaces.

The HITECH Act also funds the State Health Information Exchange Cooperative Agreement Program.[8] Under this program, the federal government has awarded $548 million to support exchange of health information across different health care organizations through the establishment of HIEs. HIEs are groups of organizations working together with a goal of improving the quality of health care delivery in a region, typically a state, by focusing on standards-based interoperability of healthcare information and healthcare information systems. The goals, capabilities, and participants in HIEs will vary across states, and participants in HIEs will vary across states. Reflecting a priority for the electronic exchange of laboratory results in HIEs. In July 2010, the ONC issued a Program Information Notice (PIN)[9] that directed HIE efforts and award grantees to focus their efforts on receipt of structured laboratory results as one of three priorities for HIEs for 2011 (the others being e-prescribing and sharing patient care summaries across organizations). HIE efforts in a laboratory, state, or region, although varying in maturity, may have relevance to laboratories that either need to or wish to participate or that see value in leveraging an HIE’s capabilities to facilitate laboratory information exchange.

## OTHER IMPLICATIONS FOR LABORATORIES OF EHR MEANINGFUL USE REQUIREMENTS

The HITECH Act and the CMS EHR incentive/penalty programs aim to increase the use of EHRs by health care providers. Greater implementation of EHRs has important clinical, operational, and business implications for laboratories, particularly those that serve physician practices. Laboratories can expect to see a dramatic increase in the expectations for LIS-EHR electronic interfaces for test results and laboratory test orders (eventually if not immediately), as physicians implement EHRs more widely. The increase in expectations for electronic interfaces will stem from (1) the fact that laboratory result interfaces will facilitate meeting meaningful use requirements (see above) for incorporation of laboratory data in an EHR and (2) the fact that implementing an EHR generally will lead to the desire to have laboratory results delivered electronically. Further to the latter point, once a physician practice has an EHR in place, the expectation will understandably follow that laboratory results will be electronically incorporated into the electronic record instead of being entered manually or instead of being viewed on separate laboratory web portal sites or the like.

Meaningful use requirements and the expected increase in EHR implementation offer some opportunities for laboratories. Implementing an electronic interface from the laboratory information system to a provider’s EHR can facilitate meeting the requirement for the incorporation of clinical laboratory test results into the EHR, and for higher volume practices, meeting the requirement is realistically possible to achieve only with an electronic interface from the laboratory (although results interfaces are not specifically mandated in the Stage 1 meaningful use criterion). Because many of the CQMs that are options for clinicians to report involve laboratory tests [Tables 5 and 6], laboratories may find opportunities to facilitate their clients’ ability to meet these meaningful use reporting requirements. At minimum, eligible providers will likely want to receive test result data in a way that will automatically populate information into their EHRs and in turn facilitate CQM reporting to meet meaningful use criteria.

### Challenges for Laboratories

For laboratories, more widespread and time-sensitive expectations for LIS-EHR interfaces present substantial challenges and bring with them considerations that go beyond the specific requirements mentioned in the Final Rules. Implementing interfaces between LISs and EHRs is not “plug and play,” and requires considerable attention to technical as well as organizational/administrative factors. In addition, there may be considerable expenses involved in implementing interfaces and maintaining interfaces. There may be lack of control or involvement available to the laboratory for EHR management at physician sites. Poor process design resulting in problems with laboratory testing may be blamed inappropriately on the laboratory.

Primary among the challenges is that the laboratory has the responsibility for the accuracy of test result data that are transmitted from the laboratory to receiving systems. The CLIA (Clinical Laboratory Improvement Amendments) regulation states this responsibility specifically (42 CFR 493.1291(a)):[10]

“The laboratory must have adequate manual or electronic system(s) in place to ensure test results and other patient-specific data are accurately and reliably sent from the point of data entry (whether interfaced or entered manually) to final report destination, in a timely manner. This includes the following:…(2) Results and patient-specific data electronically reported to network or interfaced systems.”

As part of its stated goal to promote the electronic exchange of health information and in recognition of the fact that laboratory information is an integral part of EHRs, CMS recently issued a revised guidance related to interpretation and compliance with CLIA requirements for laboratory result reporting and laboratory information exchange. This guidance was issued in March 2010 in the form of the document entitled “Issuance of Revised Survey Procedures and Interpretive Guidelines for Laboratories and Laboratory Services in Appendix C of the State Operations Manual.” [11] Generally, such interpretive guidance in the State Operations Manual is the guidance for surveyors for interpretation and application of CLIA requirements when surveying or inspecting laboratories. In the document, the CMS indicates that this is the first of a series of forthcoming memoranda from CMS on electronic exchange of laboratory information. The document provides important direction to laboratories on meeting CLIA requirements and includes revisions in requirements related to the electronic exchange of laboratory information, data retention, and management of corrected laboratory reports in EHRs. Guidance is also offered related to the definition of individuals who are authorized to receive laboratory results and how others may be designated by the authorized individuals to receive laboratory information. An extensive section of frequently asked questions includes further clarification on the above topics as well as clarification on HIEs and designating “agents” for the receipt of laboratory test results.

When meeting such requirements as the above, as well as in pursuit of a stewardship role for the quality of laboratory data in medical practice, laboratories need to be aware that EHRs may vary in their effectiveness of result display. Laboratory report elements that may be subject to variation in EHRs include:

- Reference range management.
- Explanatory comments and footnotes.
- Abnormal result flags.
- Preliminary reporting and updates.
- Reporting and documentation of corrected results.
- Unsolicited results and reflex test order/results.
- Name and address of performing laboratory.

EHR certification requirements dictate only that the CLIA-mandated elements (see previous section) that constitute a test report must be displayed in the EHRs and have no further requirements as to format, readability, or display. The issues related to the above items as well as other aspects of more complex laboratory result display commonly arise during the course of interface implementation. Failure to address these issues might have negative consequences, which include misinterpretation of laboratory results and the perception (however inappropriate it might be) that the laboratory is responsible for less than optimal display of laboratory results in EHRs and interpretive errors that might arise from such display.

Laboratories must consider other technical considerations necessary to meet the need for LIS-EHRs interfaces. Whether laboratories interface directly from their LIS to EHRs interfaces or interface through some type of integration services provider or interface engine, the capability will be necessary to interface with a wide variety of EHRs and vendors that are available. As of this writing, according to the ONC CHPL web site (http://onc-chpl.force.com/ehrcert), 115 EHRs have been certified from 79 vendors. Laboratories will need to establish a network connectivity model (e.g., virtual private network, VPN) for electronic communication with the EHR sites. Laboratories will need to secure the availability of technical support expertise for implementing and supporting the interfaces. Compatibility with ONC-mandated interoperability standards is important, perhaps not as much in the current requirements, but certainly with an eye toward the future.

### Operational Considerations for Laboratories and LIS-EHR Interfaces

Attention to the operational aspects of LIS-EHR results reporting interfaces is necessary for successful implementation and ongoing support. Some of the more important factors are summarized here:

- The importance of and methods for maintenance of the laboratory test definitions in the EHR must be understood, particularly when it comes to change control. For instance, will clients be changing their laboratory test definitions settings in the EHR, which in turn will affect the correct filing in the EHR of test results that are received in interface transmissions?
- Laboratory procedures should address change control and communication that should occur when the laboratory makes to the test definition in its own LIS. Some of these changes, such as test definition updates or reference range adjustments, may impact interface transmission and/or the display of results in the receiving EHR and, therefore, procedures are necessary for communicating such changes to the interfaced sites.
- Ongoing troubleshooting of interfaces and interface-related client support issues will grow as the number of interfaces grow, and must be accounted for in laboratory management planning.
- Overall client site contact and engagement will be important to be able to have successful communication of laboratory results electronically on an ongoing basis and to manage changes. Implementing EHRs interfaces requires some involvement by support personnel at the client site; however, getting access to and attention of such people at client sites can be challenging and frustrating if such personnel resources at any particular site even exist.
- There may be a need to train EHR interface clients as to how laboratory results are viewed.
- A process for handling and communicating corrected results must be implemented and validated.
- Depending on the practice setting, the establishment of procedures may be necessary for the communication of laboratory test results in situations in downtime situations when the interface is not available.
- Providers with differing clinical needs, different test mixes, and/or different EHR solutions may require different laboratory workflow or at least must be accommodated within laboratory operations; however, this need must be balanced against reduced efficiency that may result from creating too many exceptions or variations in laboratory procedures.
- LIS upgrades or updates must also take into account any effects on interfaced systems and sites.

### Anticipated Requirements in Future Stages of Meaningful Use

While current requirements for Stage 1 of meaningful use criteria and related data standards adopted by HHS/ONC are relatively limited with regard to the laboratory, anticipated requirements in the future stages of meaningful use can be expected to have a greater impact on the laboratory. As mentioned, CPOE as a requirement for laboratory test orders was removed from Stage 1, but is expected to be present in Stage 2. When implemented, electronic orders for laboratory tests will originate in the CPOE module of EHRs and will be subject for the vagaries of how CPOE is implemented in different EHRs. Laboratories should expect to have processes in place to handle electronic transmission of orders from interfaced clients in advance and expectation of these requirements. Test menu/test catalog management will be of paramount importance, given the diversity of the EHR environment (as evidenced by the number of EHRs already certified). CPOE systems must be configured correctly for laboratory test ordering in terms of menus, order tests, and the options for how test order choices are presented to the ordering physician. Test requests in CPOE systems must include the capability to include all the items that CLIA mandates in test requests and, furthermore, the CPOE systems should be set up to accommodate other nuances of laboratory test ordering, including “ask at order entry” questions and provision of clinical information when necessary. There are significant negative consequences for the laboratory of improperly designed or implemented EHR CPOE processes (even if the laboratory has little influence or opportunity for involvement in the implementation process), such as incorrect, incomplete, and/or inappropriate test orders as well as inefficiencies owing to CPOE problem resolution. Depending on the clinical setting, the CPOE process for laboratory tests may need to account for future orders, duplicate order handling, and canceled order handling. Billing problems may arise as well if not factored into planning.

Stage 2 meaningful use criteria are expected to include requirements for electronic transmission of diagnostic test results that extend beyond the current applicability to numeric results and yes/no results and also include pathology results and genetic tests (in addition to radiology, cardiac imaging, pulmonary function tests, etc.).

As HHS has adopted LOINC and HL7 v2.5.1 in certain EHR certification criteria, it might be expected that broader requirements regarding use of these data standards may be forthcoming in future stages. LOINC, and the capabilities of laboratory information systems to accommodate LOINC, is of particular interest for laboratory test information management as laboratories examine the capabilities of their LISs. In line with the HHS-stated goals, broadly speaking, there will be greater expectations for exchange of healthcare data with unaffiliated entities and more decision support in general, both of which can be expected to involve laboratory tests.

## CONCLUSIONS

As a result of the recent federal government efforts under the HITECH Act, most notably EHR meaningful use and EHR certification criteria, use of EHRs can be expected to increase dramatically in the coming months and years. Meaningful use criteria dictate how eligible providers and hospitals must use EHRs technology, while certification standards specify what capabilities that EHR must possess in order to support meaningful use. Some of these criteria and requirements are directly applicable to laboratory testing currently, and more promise to be applicable to laboratory testing in the future.

As EHR uptake expands, there will be greater expectations for electronic interchange of laboratory information, and laboratories must prepare now to meet the needs of the future environment. Implementation of LIS-EHR interfaces promises to be a major priority in the future and a challenge for laboratories serving outreach clients. In addition, some of the new and future requirements and programs may provide other opportunities for ways that laboratories can better serve their provider community.
